# Study on the Mechanism of Lipid Peroxidation Induced by Carbonate Radicals

**DOI:** 10.3390/molecules29051125

**Published:** 2024-03-02

**Authors:** Heng Cao, Sheng-Feng Xiong, Li-Long Dong, Zhou-Tong Dai

**Affiliations:** 1 Department of Gynecological Oncology, Tongji Hospital, Tongji Medical College, Huazhong University of Science and Technology, Wuhan 430030, China; 2 National Clinical Research Center for Obstetrics and Gynecology, Cancer Biology Research Center (Key Laboratory of the Ministry of Education), Tongji Hospital, Tongji Medical College, Huazhong University of Science and Technology, Wuhan 430030, China; 3 Department of Pharmaceutical Analysis, School of Pharmacy, Hebei Medical University, Shijiazhuang 050031, China

**Keywords:** carbonate radical, DFT, lipid peroxidation, hydrogen abstraction, (3Z,6Z)-nona-3,6-diene

## Abstract

Based on the reported research, hydroxyl radicals can be rapidly transformed into carbonate radicals in the carbonate–bicarbonate buffering system in vivo. Many of the processes considered to be initiated by hydroxyl radicals may be caused by carbonate radicals, which indicates that lipid peroxidation initiated by hydroxyl radicals can also be caused by carbonate radicals. To date, theoretical research on reactions of hydrogen abstraction from and radical addition to polyunsaturated fatty acids (PUFAs) of carbonate radicals has not been carried out systematically. This paper employs (3Z,6Z)-nona-3,6-diene (NDE) as a model for polyunsaturated fatty acids (PUFAs). Density functional theory (DFT) with the CAM-B3LYP method at the 6-311+g(d,p) level was used to calculate the differences in reactivity of carbonate radicals abstracting hydrogen from different positions of NDE and their addition to the double bonds of NDE under lipid solvent conditions with a dielectric constant of 4.0 (CPCM model). Grimme’s empirical dispersion correction was taken into account through the D3 scheme. The energy barrier, reaction rate constants, internal energy, enthalpy and Gibbs free energy changes in these reactions were calculated With zero-point vibrational energy (ZPVE) corrections. The results indicated that carbonate radicals initiate lipid peroxidation primarily through hydrogen abstraction from diallyl carbon atoms. The reaction of hydrogen abstraction from diallyl carbon atoms exhibits the highest reaction rate, with a reaction rate constant approximately 43-fold greater than the second-ranked hydrogen abstraction from allyl carbon atoms. This process has the lowest energy barrier, internal energy, enthalpy, and Gibbs free energy changes, indicating that it is also the most spontaneous process.

## 1. Introduction

Reactive oxygen species (ROS) are highly active oxygen-containing molecules that are generated as natural byproducts of cellular metabolism [1]. These molecules form within cells due to the partial reduction of oxygen, resulting in unstable compounds like superoxide anion (•O_2_^−^), hydrogen peroxide (H_2_O_2_), and hydroxyl radicals (•OH) [2]. They can also be generated in response to external stressors such as radiation, pollutants, and toxins. Excessive ROS production can lead to oxidative stress, an imbalance between ROS production and antioxidant defenses. This imbalance contributes to diseases like cancer [3], cardiovascular disease [4], and neurodegenerative diseases [5], along with cellular dysfunction and aging [6].

ROS can react with intracellular lipids, proteins and nucleic acids to lead to the destruction of cell structure and function [7]. Among these, lipid peroxidation is a critical factor in cell damage [8]. It is a chemical process occurring when ROS like superoxide radicals, hydrogen peroxide, and hydroxyl radicals attack and oxidize unsaturated fatty acids in cell membranes [9]. Specifically, ROS can attack and remove an electron from a fatty acid molecule located in a cell membrane, generating fatty acid radicals. These radicals then react with molecular oxygen to produce peroxyl radicals [10]. Peroxyl radicals interact with other fatty acid molecules, propagating the lipid peroxidation chain reaction [11], forming reactive lipid peroxides. These peroxides can harm cell components, including membranes and proteins.

The initiation reaction of lipid peroxidation is the first step in the process and involves the formation of a lipid radical from a fatty acid or a phospholipid molecule. It can be initiated in several ways, including exposure to free radicals, ionizing radiation [12], or enzymatic reactions [13].

As the most active member of reactive oxygen species, hydroxyl radicals play a crucial role in initiation of lipid peroxidation. Hydroxyl radicals react with almost all biomolecules at or near diffusion-controlled rates [14]. Therefore, any ·OH formed within the body will react with any substance present at its formation site, making it difficult to directly capture and demonstrate its formation in biological systems [15]. Hydroxyl radicals can react quickly with residues of polyunsaturated fatty acids (PUFAs) of cell membranes, thiol-containing proteins and nucleic acids [16]. Hydroxyl radicals can initiate lipid peroxidation by abstracting a hydrogen atom from the fatty acid or phospholipid molecule, leading to the formation of a lipid radical and a new water molecule. This process can be summarized as:

RH + ·OH → R· + H_2_O (1)

where RH is the fatty acid or phospholipid molecule.

In recent years, with the progress of detection methods and the development of free radical theory, it is believed that in the presence of a carbonate–bicarbonate buffer system in the body, once a hydroxyl radical is generated, it will quickly react with carbonate and bicarbonate radicals and transform into carbonate radicals (the bicarbonate radical is easily ionized into carbonate radicals and hydrogen ions) [17,18]. Many of the processes previously thought to be caused by hydroxyl radicals may be carried out by carbonate radicals [19,20].

In addition to deriving from hydroxyl radicals, carbonate radical anions can also be formed through reactions involving peroxynitrite (ONOO^−^), a potent ROS formed by the rapid reaction between superoxide and nitric oxide (·NO). The reaction of peroxynitrite with carbon dioxide leads to the generation of carbonate radicals [21]:

ONOO^−^ + CO_2_ → ONOOCO_2_^−^ → •CO_3_^−^ + •NO_2_ (2)

Moreover, the carbonated radical can be produced by an enzyme-catalyzed reaction, which can be produced in the bicarbonate buffer system containing Cu, Zn superoxide dismutase and H_2_O_2_ [22]. The reaction is:

SOD-Cu(II)•OH + HCO_3_^−^ → SOD-Cu(II)+ •CO_3_^−^ + H_2_O (3)

Carbonate radicals are so prevalent in the body that their harm is gradually being paid attention to [23]. It is generally believed that although the carbonate radical is not as active as the hydroxyl radical, it is still a very active free radical [22], which can react with nucleic acids, proteins, and steroids [24,25,26,27,28,29]. Carbonate radicals can cause direct DNA damage by oxidizing nucleotide bases, leading to mutations and genomic instability [30]. This damage can result in impaired cellular functions, genomic alterations, and an increased risk of cancer development [24]. Carbonate radicals can also target amino acid residues in proteins, leading to protein oxidation and modifications [26]. Oxidized proteins can lose their structure and function, disrupting vital cellular processes and potentially triggering aging and inflammatory responses [31].

Although carbonic radicals are considered to be more prevalent in vivo than hydroxyl radicals, because the carbonate radical is an ionic free radical, it is thought that the carbonate radical is unlikely to be an efficient initiator of lipid peroxidation [23]. Concerning the potential reaction of carbonate radicals with polyunsaturated fatty acids (PUFAs), there is still a notable lack of direct experimental evidence in this area. Only the study conducted by Buehl et al. has examined the reaction between carbonate monomethyl ester radicals (neutral radicals) and PUFAs [29]. Despite the limited solubility of carbonate radical anions in low polarity lipid solvents, their high reactivities and the huge surface area of cell membranes within the body provide certain opportunities for carbonic radicals to trigger lipid peroxidation. There are many other places in the body, such as blood, liver, heart, exercising muscles, and adipose tissue, where free fatty acids are abundant and they also have ample opportunity to contact with carbonate radicals. Hence, even though direct experimental research on carbonate radical-induced lipid peroxidation is lacking, it is necessary to explore the feasibility of lipid peroxidation induced by carbonate free radicals theoretically. However, there is no detailed quantum chemistry calculation study in this aspect. Therefore, in this paper, the hydrogen abstraction of carbonate radical from different types of hydrocarbon groups of PUFA with (3Z,6Z)-nona-3,6-diene as a model was explored using the density functional quantum chemistry calculation method with gaussian09 package.

## 2. Results

### 2.1. Reaction of Carbonate Free Radical with Polyunsaturated Fatty Acids

**Figure 1:**
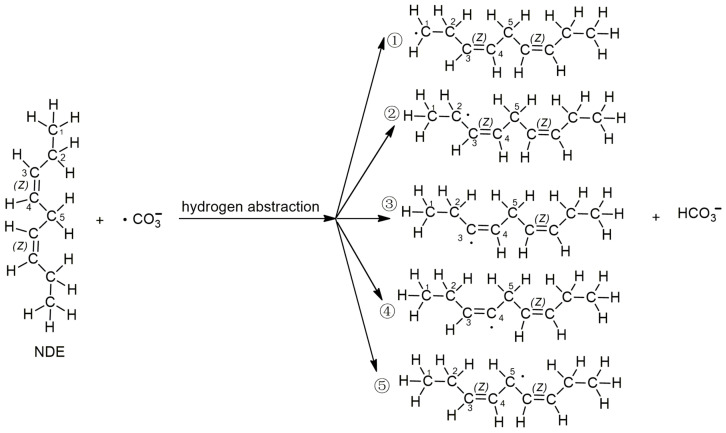
The reactions of the carbonate radical to abstract hydrogen from different positions of NDE (1, 2, 3, 4 and 5) shown as reactions ①, ②, ③, ④ and ⑤.

It is well known that the main target of lipid peroxidation is polyunsaturated fatty acids. Linoleic acid is a typical representative of polyunsaturated fatty acids in lipids. Many researchers use linoleic acid as a reactant to study lipid peroxidation [32,33]. And the initial lipid model employed in the computational chemistry study is (3Z,6Z)-nona-3,6-diene (NDE) [34,35], which represents the unsaturated fragment of linoleic acid with 2 double bonds (Figure 1). Since both carbonated radicals and hydroxyl radicals are active radicals, the reported lipid peroxidation induced by active radicals is mainly caused by hydrogen abstraction by free radicals. In this study, the hydrogen abstraction reactions of carbonated radicals from different sites of NDE were first calculated. The reactions of the carbonate radical to abstract hydrogen from different positions of NDE (1, 2, 3, 4 and 5) are shown in Figure 1.

**Figure 2:**
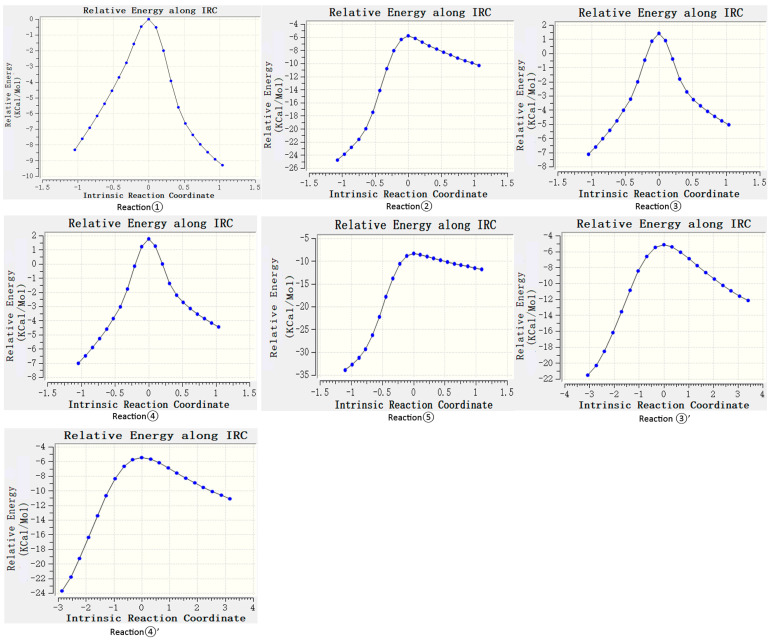
IRC profiles of hydrogen abstractions reactions ①, ②, ③, ④ and ⑤ and carbonate radical addition reactions ③′ and ④′.

**Figure 3:**
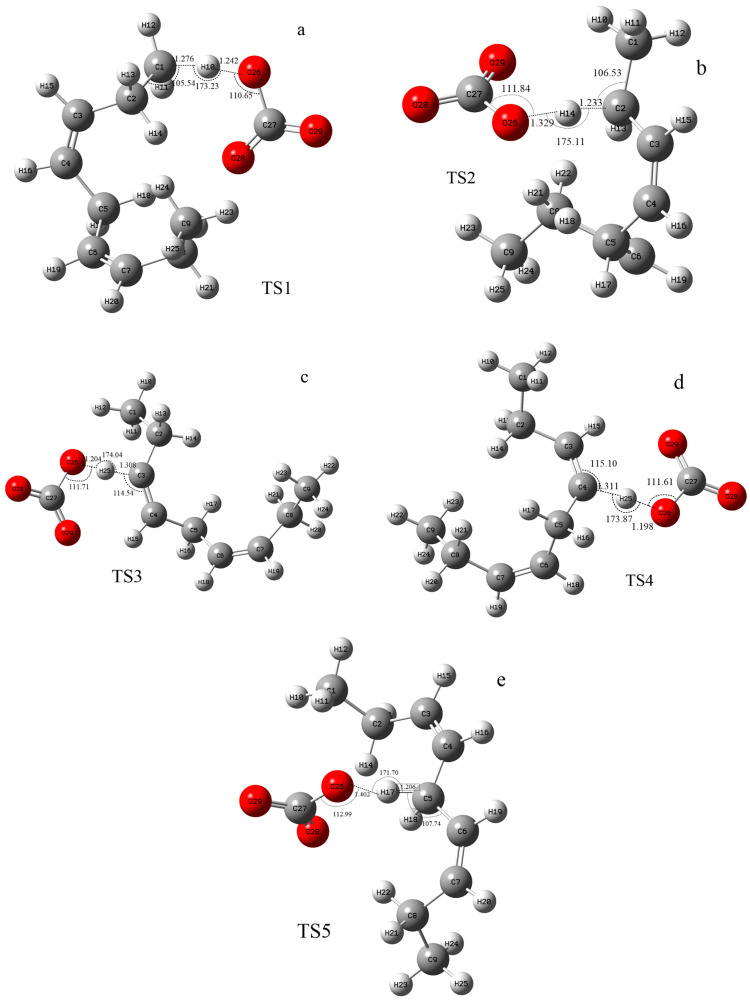
The structure of the transition states of the carbonate radical to abstract hydrogen from different positions of NDE. ((**a**–**e**) are the images of transition states of carbonate radical abstracting hydrogen from C1, C2, C3, C4 and C5 respectively). The calculation was carried out at UCAM-B3LYP/6-311+g(d,p) with the CPCM solvent model and ε = 4.0 (angle unit: degree; distance unit: Å).

According to the difference in the activity of hydrogen abstraction, the hydrogens on the C-H bonds in the polyunsaturated fatty acids of the lipid can be mainly divided into four kinds, the hydrogens on the carbon atoms that are not adjacent to the double bond (isolated C-H such as position 1 in Figure 1), the hydrogens on mono-allylic carbon (position 2), alkenyl bond carbon (positions 3 and 4) and bis-allylic carbon (position 5). Due to the different environments of the hydrogens in the locations described above, their reactivities are also different. In this study, the reaction processes of hydrogen abstraction from different carbon atoms by carbonate radicals were calculated. The transition states of the reaction of carbonic radical to abstract hydrogen from different types of carbon atoms were obtained using the TS method structure optimization in Gaussian09 with UCAM-B3LYP/g-311+g(d,p) levels and the CPCM solvent model (ε = 4.0). The transition states (TSs) were confirmed by frequency and IRC calculations at the same level as the structure optimization. The only one imaginary frequency was obtained for each reaction of this study and shown in Table 1. The IRC profiles of hydrogen abstractions reactions ①, ②, ③, ④ and ⑤ and carbonate radical addition reactions ③′ and ④′ are displayed in Figure 2. Figure 3a depicts the transition state of the carbonate radical abstracting hydrogen from an isolated C-H bond (TS1). It can be observed that the carbonate radical O26 abstracts H10 from the C1 atom of the NDE molecule. The bond length of O26-H10 is 1.242 Å, while the bond length of C1-H10 is 1.276 Å, and the ∠O26-H10-C1 angle is 173.23°. ∠H10-C1-C2 is 105.54° and ∠H10-O26-C27 is 110.65°, respectively. Figure 3b shows the transition state (TS2) in which carbonate radical abstracts hydrogen from the mono-allylic carbon (C2). The bond lengths of O26-H14 and C2-H14 are 1.329 Å and 1.233 Å, respectively. Figure 3c,d show the structure of the transition states of hydrogen abstraction from the alkenyl bond carbons (C3 and C4). In TS3, the distances of transferred hydrogen H25 to O26 of the carbonate radical and C3 of NDE are 1.204 Å and 1.308 Å, respectively. In TS4, the bond lengths of O26-H19 and C4-H19 are 1.198 Å and 1.311 Å, respectively. For the transition state (TS5) of hydrogen abstraction from bis-allylic carbon (C5), the O26-H17 distance is 1.402 Å, while the C5-H17 bond length is 1.206 Å (Figure 3e). It can be observed that in the transition states of hydrogen abstraction from saturated carbon atoms such as C1, C2 and C5 (Figure 3a,b,e), the transferred hydrogen atoms are closer to the O26 of the carbonate radical than to the carbon atoms of the NDE molecule. On the contrary, the transferred H atom is closer to the original C atom of the double bond of NDE (Figure 3c,d).

**Figure 4:**
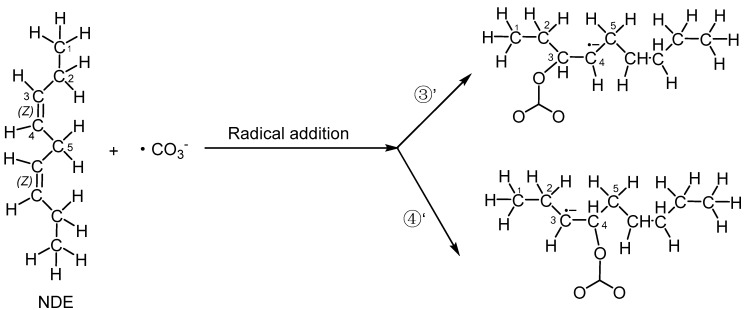
The addition reactions of the carbonate radical to C3 and C4 positions of NDE (reactions ③′ and ④′).

**Figure 5:**
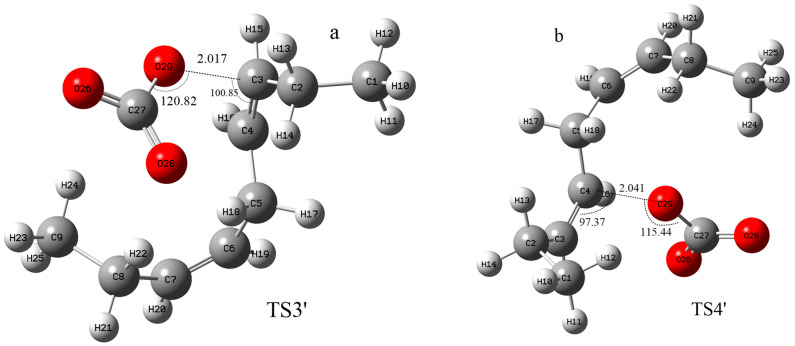
The structure of the transition states for the addition of carbonate radical to C3 and C4 positions of NDE (TS3′ (**a**) and TS4′ (**b**)). The calculation was carried out at UCAM-B3LYP/6-311+g(d,p) with the CPCM solvent model and ε = 4.0 (angle unit: degree; distance unit: Å).

In addition to hydrogen abstraction, hydroxyl radicals can also be added to the olefinic bonds of polyunsaturated fatty acids [36]. Can carbonate radicals also be added to polyunsaturated fatty acids? In order to investigate the possibility of the addition reaction between carbonate free radicals and polyunsaturated fatty acids, the transition states (TS3′ and TS4′) for the addition of carbonate free radicals to C3 and C4 atoms of NDE (reactions ③′ and ④′ are shown in Figure 4), respectively, were calculated and their structures are shown in Figure 5. It can be seen that in the transition states of TS3′ and TS4′, the distances of the O29 of carbonated radical to the C3 and C4 of NDE are 2.017 Å and 2.041 Å, respectively. and the angles between C3-O29 (C4-O29) and O29-C27 of the carbonate radical is 120.82° (115.44°).

### 2.2. The Potential Energy Surfaces of the Reactions between Carbonate Radicals and Different Positions in NDE

**Figure 6:**
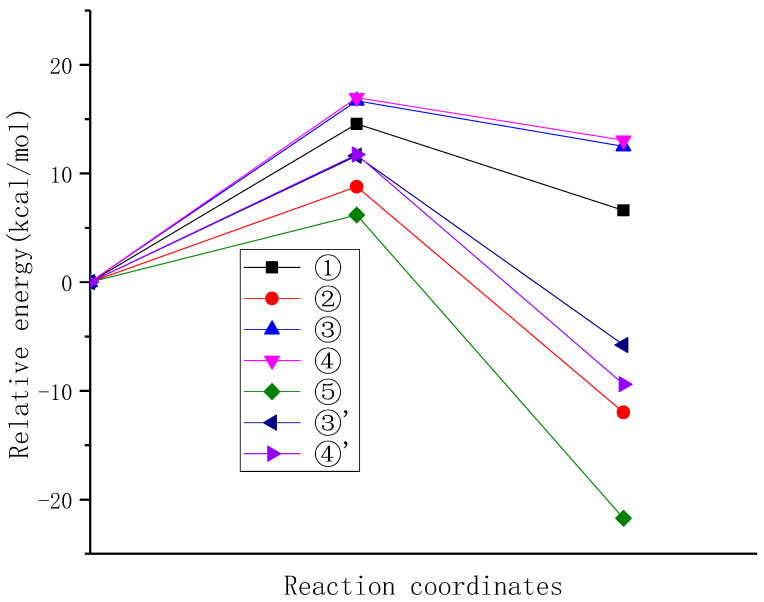
The potential energy surfaces of the reaction of carbonate radical with NDE at different positions.

In order to compare the reactivity differences of different positions of NDE in reaction with carbonate radicals, the energy barrier changes in the reaction process were further analyzed. As shown in Figure 6, it can be observed that the highest energy barriers are associated with the hydrogen abstraction from the double-bonded carbon atoms (C3 and C4) by the carbonate radical (reactions 3 and 4), which are 16.688 kcal/mol and 16.975 kcal/mol, respectively. The next highest energy barrier is observed for the hydrogen abstraction from the isolated carbon atom (C1) (reaction 1), with an energy barrier of 14.573 kcal/mol. On the other hand, the lowest energy barriers are observed for the hydrogen abstraction from the mono-allylic carbon (reaction 2) and the diallylic carbon (reaction 5), particularly for the hydrogen abstraction from the diallylic carbon of reaction 5, with an energy barrier of 6.186 kcal/mol, only 36.48% that of reaction 4. The reaction order by energy barriers from high to low is ④ > ③ > ① > ② > ⑤, which indicates that diallyl hydrogen (position 5) is most easily abstracted, and it is the preferred reaction position for the hydrogen abstraction by carbonate radicals, followed by allyl hydrogen, which is consistent with the hydrogen abstraction selectivity of hydroxyl radicals [37].

In addition to the hydrogen abstraction reaction of carbonate radicals, the addition reaction of carbonate radical to NDE was also investigated, and the energy barriers of the addition of carbonated radical to double-bonded carbon atom of NDE were calculated. The energy barriers of the addition of carbonate radical to C3 and C4 atoms of NDE are basically the same, which are 11.632 kcal/mol and 11.740 kcal/mol, respectively. Although the energy barrier of addition reaction is smaller than that of hydrogen abstraction from double bond and isolated carbon atoms, it is still higher than that of hydrogen abstraction from mono-allyl and diallyl carbon atoms. Therefore, from the view point of energy barrier, the reactivity of the addition reaction of carbonate radical to the double bond is weaker than that of the hydrogen abstraction from diallyl and allyl carbon atoms. This aligns with the trend observed in the allylic H-abstraction by the hydroxyl radical reaction from propene conducted by Szori et al., where the allylic hydrogen abstraction reaction is more favorable compared to the addition reaction of hydroxyl radicals to the double bond [38].

### 2.3. Electron Spin Density Analysis

**Figure 7:**
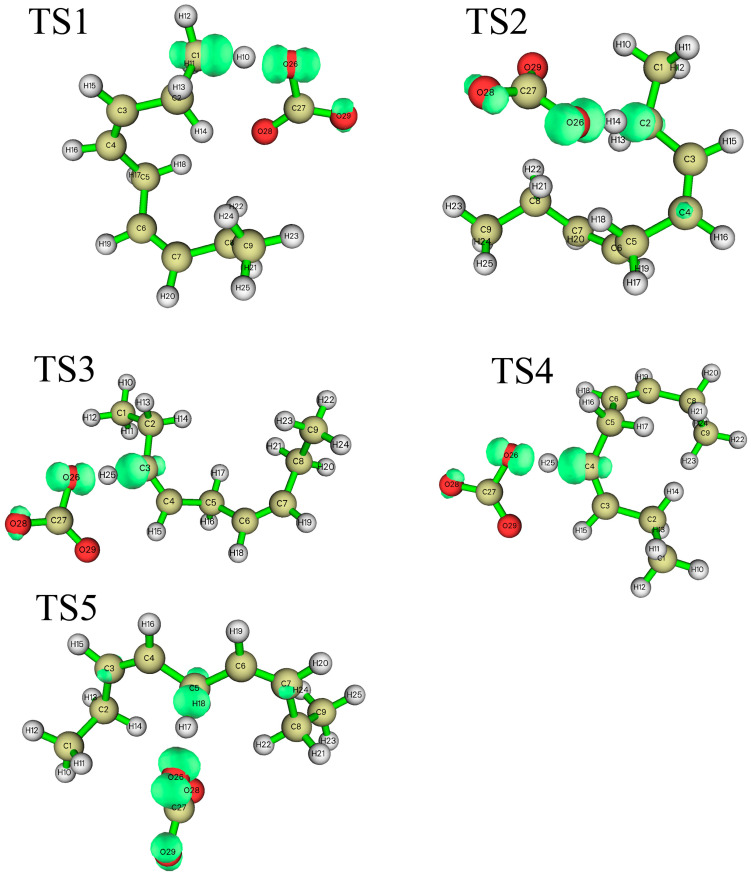
Spin density distribution of transition states 1–5 (TSs 1–5).

Multiwfn [39] was used to analyze the spin density of the transition states optimized as described previously. The electron spin density distributions of the five transition states of hydrogen abstraction reaction were shown in Figure 7. It can be seen that the electron spin density in the transition states of hydrogen extraction reactions ①–⑤ is mainly distributed on the oxygen atom (O26) in hydrogen abstraction and the carbon atoms that lose hydrogen atoms. It is shown that along with the transfer of hydrogen atoms, the electron spin (free radical) is also transferred (from the carbonate radical to NDE), so the process is a hydrogen transfer process (HAT) [40].

**Figure 8:**
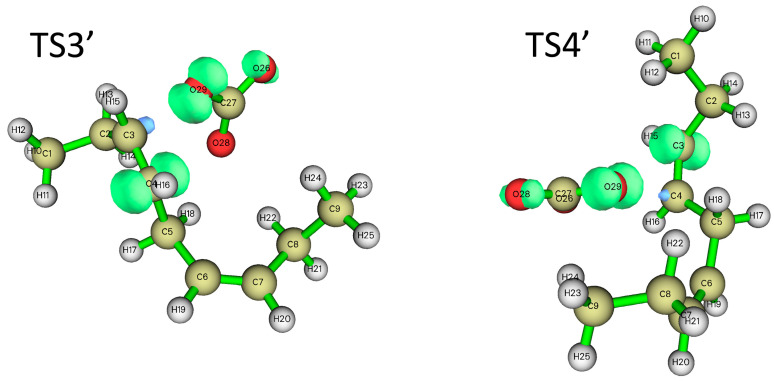
Spin density distribution of transition states 3′ and 4′ (TSs 3′ and 4′).

Figure 8 shows the electron spin density distribution in the transition states (TS3′ and TS4′) of the double bond addition reaction between carbonated radical and NDE. It can be seen that as the carbonate radical approaches one of the carbon atoms in the double bond, its spin density (radical) component is also transferred to the other carbon atom in the double bond. It is shown that as the carbonate radical gradually forms a covalent bond with one carbon atom of the NDE double bonds, its spin (radical) component is transferred to the other carbon atom of the original double bond.

### 2.4. The Reaction Rate Constant

In order to compare the reaction rates of hydrogen abstraction and carbonated radical addition at different sites of NDE described above, the rate constants (k) of the reactions of hydrogen abstraction ①–⑤ and addition of carbonated radical ③′ and ④′ were calculated using Equation (3), as shown in Table 1. It can be seen that reaction 5 (carbonate radical abstraction of hydrogen from diallyl carbon atoms) has the fastest reaction rate, and its rate constant k is 5.232 × 10^−21^ s^−1^/(mole/cm^3^), which is followed by the hydrogen extraction of from allyl carbon atoms (reaction 2) and the addition of carbonate radicals (reactions 4′ and 3′), with k values of 1.208 × 10^−22^ s^−1^/(mole/cm^3^), 1.375 × 10^−25^ s^−1^/(mole/cm^3^) and 1.078 × 10^−25^ s^−1^/(mole/cm^3^), respectively. As comparison, the reaction rates of hydrogen abstraction on other sites, such as reactions ①, ③ and ④, can be ignored. The rate constant of the reaction ③ is slightly higher than that of the reaction ④. For reaction ⑤, the rate constant is approximately 43-fold that of the second fast reaction ②. Therefore, lipid peroxidation mainly occurs on diallyl carbon atoms, which is consistent with the usual results of lipid peroxidation.

### 2.5. The Energy Change in the Reactions

Table 2 shows the internal energy, enthalpy and Gibbs free energy changes in the reactions ①–⑤, ③′ and ④′. It can be seen from Table 2 that the internal energy change (${\Delta U}_{298K}^{{}^{\circ}}$) and enthalpy change (${\Delta H}_{298K}^{{}^{\circ}}$) of hydrogen abstraction reactions from isolated carbon atoms (reaction 1) and double-bonded carbon atoms (reactions ③ and ④) are greater than 0 kcal/mol, indicating that they are endothermic reactions, while the values of ${\Delta U}_{298K}^{{}^{\circ}}$ and ${\Delta H}_{298K}^{{}^{\circ}}$ of the other reactions are negative, showing that they are exothermic reactions.

Table 2 also lists the change in Gibbs free energy (${\Delta G}_{298K}^{{}^{\circ}})$ of the reactions. It can be seen that except for the reaction of hydrogen abstraction from diallyl and allyl carbon atoms, the changes in Gibbs free energy of other reaction processes are positive, indicating that the reaction of hydrogen abstraction from diallyl and allyl carbon atoms by carbonate radicals is a thermodynamic spontaneous process, which is easier to carry out than other reactions.

## 3. Discussion

There have been some computational investigations on the mechanism of lipid peroxidation initiation. Szori et al. compared the H-abstraction reactivity from different positions of 1,4-type polyalkenes as a model for free radical trapping by PUFAs with the G3MP2//BH&HLYP computational method [37]. The room temperature rate constants were calculated with transition state theory and it was found that the hydrogen abstraction from the bis-allylic atom by ·OH is fastest, followed by mono-allylic atom. Tejero et al. explored the ·OH-radical-induced mechanism of lipid peroxidation, involving hydrogen abstraction followed by the addition of O_2_ using at CPCM-MPWB1K/MG3S//MPWB1K/6-31+G(d,p) level [34]. It is found that when the whole linoleic acid is considered as the model in the calculations, hydrogen abstraction from the bis-allylic C11 atom becomes the most favorable, in good agreement with experiment, showing the potential of quantum chemical computation in exploring the mechanism of lipid peroxidation.

Szori et al. [41] studied the nonenzymatic pathway of polyunsaturated fatty acid oxidation by first-principle calculation of the reactions of ·OH radical with 1,4-pentadiene and arachidonic acid. Employing the 1,4-pentadiene + ·OH reaction system, they found that the terminal and nonterminal additions and the indirect hydrogen abstraction reaction have pseudo-negative activation enthalpies due to the pre-reaction complex. The H-abstraction is found to be the most exothermic reaction among those studied (−167.9 kJ·mol^−1^).

It has been reported that in the carbonate–bicarbonate buffering system, hydroxyl radicals can be converted into carbonate radicals, and many hydroxyl-radical-induced processes may be carried out through carbonate radicals [17]. Also, carbonate radicals can be derived of peroxynitrite and an enzyme-catalyzed reaction in the bicarbonate buffer system containing Cu, Zn superoxide dismutase and H_2_O_2_ [22]. Considering the living body is a carbonate–bicarbonate buffer system, whether lipid peroxidation can be initialized by carbonate radicals still lacks systematic experimental and theoretical research. Therefore, the theoretical evaluation of the initiation of lipid peroxidation by carbonate radicals through quantum chemical calculation is a good guidance for the experimental research in this field. However, the selection of different quantum chemical calculation functionals has an important impact on the accuracy of the calculation results, so it is necessary to select appropriate calculation functionals before the calculation on the reactions of carbonate radicals and polyunsaturated fatty acids. In order to identify the optimum DFT method, Buhl et al. [29] carried out quantum chemical calculation studies on the changes in absolute energy for 12 reactions using the G4 high-precision calculation method as the gold standard. They have compared the mean absolute deviations (MADs) and root mean square deviations between the 23 DFT function (including MP2 ab initio method) and the G4 dataset. The 12 reactions chosen included deprotonations, ionizations, H-atom abstractions, radical additions to propene and β-scissions. The 23 DFT function compared include CAM-B3LYP, BLYP, B1LYP, B3LYP, M05, M052X, M06, M062X, ωB97 and PBEPBE. These comparisons indicated that the CAM-B3LYP function, which combines the hybrid qualities of B3LYP with the long-range correction proposed by Tawada et al. [42] gave the best results in comparison with G4 method. Thus, in this study, the CAM-B3LYP function was employed to conduct the computation of the initiation of lipid peroxidation by carbonate radicals. On the other hand, using actual molecules as a computing system can make the results closer to the real situation. However, due to the limitations of actual computing power and resources, for large multi-atom systems, it is often necessary to use a lower-level algorithm and baseset to complete the calculation, which increases the calculation error on the reaction site of concern. Since saturated fat chain has little influence on the general reaction site of PUFAs, and in practice, lipid peroxidation rarely initialized from the position of the saturated fat chains, thus, NDE molecules containing the typical characteristics of unsaturated fatty acids (diallyl hydrogen, allyl hydrogen, alkenyl bonded hydrogen and hydrogen on isolated carbon) were used as the calculation model in this study, and a higher-level 6-311+g(d,p) baseset was used for calculation. Energy barriers and rate constants for hydrogen abstraction reactions from various carbon atoms of NDE, as well as carbonate radical addition to double bonds, were calculated. For the influence of solvent, they adopted a CPCM solvent model, which was used in many calculation studies of lipid peroxidation or carbonate free radicals [18,34,43]. Therefore, the CPCM solvent model was used in this study to calculate the reaction of lipid peroxidation initiation by carbonate free radicals. The CPCM solvent model at ε = 4.0 was employed to simulate lipid solvent conditions. Additionally, Grimme’s empirical dispersion correction was considered with the keyword “empiricaldispersion=GD3”. Zero-point vibrational energy (ZPVE) corrections were employed for the energy calculation of each stationary point. It was found that the order of different reaction rates was ④ < ③ < ① < ④′ < ③′ < ② < ⑤. In terms of the energy change in the reactions, hydrogen abstraction reactions from isolated carbon atoms (reaction ①) and double-bonded carbon atoms (reactions ③ and ④) are endothermic reactions, but the others are exothermic reactions. It is found by calculating the Gibbs free energy change in the reaction that the reactions of hydrogen abstraction from diallyl and allyl carbon atoms by carbonate radicals are thermodynamic spontaneous. In general, the hydrogen abstraction from diallyl carbon atoms is the most favorable way for carbonate radicals to trigger lipid peroxidation. This is consistent with the experimental results that all lipid peroxidation occurs preferentially in diallyl carbon atoms under physiological conditions [44], although there is no direct evidence of lipid peroxidation induced by hydrogen abstraction of carbonate radicals.

Under the condition of a bicarbonate/carbonate buffer system, many of the processes previously thought to be caused by hydroxyl radicals may be carried out by carbonate radicals [19,20]. However, direct experimental evidence on whether carbonic radical can induce lipid peroxidation is still lacking. Epperlein et al. [45] studied the process of bicarbonate hemodialysis and found that carbonate radical and formic acid free radicals generated under high bicarbonate concentration might be one of the causes of accompanying lipid peroxidation. With the calculation in this study, it is confirmed theoretically that carbonate radical can not only initiated lipid radical by abstracting hydrogen from the fat chain of PUFAs, but also preferentially abstract hydrogen from allyl carbon atoms. Generally, the site selectivity and reactivity order of lipid peroxidation initialized by carbonate radicals are of great significance for the understanding and prevention of lipid peroxidation induced by different stresses in vivo. Of course, the conclusions of this study need to be confirmed by further experimental results.

## 4. Computational Methods

### 4.1. Quantum Chemistry Computation

Since the double bonds in natural polyunsaturated fatty acids predominantly exist in the cis configuration, (3Z,6Z)-nona-3,6-diene(NDE) was used as a model for the study of the hydrogen abstraction of carbonate radical from different types of hydrocarbon groups of PUFA. Spin-restricted and spin-unrestricted DFT calculations were conducted using Gaussian09 D01 software [46]. The UCAM-B3LYP function of DFT, known for its reproducibility of high-level ab initio benchmarks best [47] and for its wide application in radical reaction studies [29], was chosen to study decarboxylation and deprotonation reactions at 6-311+g(d,p) level. Grimme’s empirical dispersion correction with the keyword “empiricaldispersion=GD3” was adopted. Vibration analyses were performed to identify stationary points, distinguishing reactants, and products (without imaginary frequencies), as well as transition states (with one imaginary frequency). Intrinsic reaction coordinates (IRC) were used to confirm the transition state connecting the expected reactant and product (or intermediate). The conductor-like polarizable continuum model (CPCM) was used to account for solvent effects [48]. For the CPCM calculations, an ε value of 4.0 was employed for the dielectric constant, to simulate the hydrophobic interior of a lipid membrane [34]. All calculations were conducted at the ground state, and zero-point vibrational energy (ZPVE) corrections were applied for the energy calculation of each stationary point. The initial structures of reactants, transition states, and products were prepared using ChemBioDraw Ultra 12.0.

### 4.2. Rate Constant Computation

The energy barriers of the reactions were determined by calculating the energy difference between the transition states and the corresponding reactants with the zero-point correction. The internal energy change (${∆U}_{298K}^{{}^{\circ}})$, enthalpy change ($∆H_{298K}^{{}^{\circ}}$) and Gibbs free energy change ($∆H_{298K}^{{}^{\circ}}$) of the reactions were obtained by comparing the internal energy, enthalpy and Gibbs free energy of the products and reactants with the zero-point correction. To calculate the rate constant (*k*) of the reactions, transition state theory was employed, and the following equations were used [49,50,51]:

(4) $$\kappa =1+{\left(\frac{h}{k_{b}\cdot T\cdot \zeta_{fre}\cdot 3\times 10^{10}}\right)}^{\frac{2}{24}}$$

(5) $$k=\frac{\kappa \cdot \sigma \cdot k_{b}\cdot T}{h}\cdot \frac{k_{b}\cdot T}{P}\cdot 10\cdot \frac{Q_{TS}}{Q_{A}\cdot Q_{B}}\cdot e^{\left(\frac{-∆E}{R\cdot T\cdot 1000}\right)}$$

In the above equations, *P*, *T*, *h*, *R* and $k_{b}$ are physical constants. *P* and *T* are the pressure (bar) and reaction temperature (298 K), respectively. *h* is the Planck constant (6.63 × 10^−34^ J·s). R is the molar gas constant (8.314 J/(mol·K)) and $k_{b}$ is the Boltzmann constant (equels to 1.38 × 10^−23^ J·K^−1^). σ is the degree of degeneracy of the reaction path (σ = 1 in this study). ∆E denotes the static potential threshold on the minimum energy response path (MEEP). $Q_{TS}$ is the partition function of the transition state. $Q_{A}$ and $Q_{B}$ are the partition functions of the reactants [52]. $\zeta_{fre}$ signifies the imaginary frequencies of the transition state. κ is the tunnel effect correction factor, which can be calculated by the Equation (4). Equation (5) is used to calculate the reaction rate. *N*_A_ represents the Avogado constant. All the 3D molecular structures were made by GaussView 5.0 [53].

## Figures and Tables

**Table 1:** The corresponding parameters of reaction rate constant calculation for the reactions of carbonate radical with NDE on different position (298.15 K).

Reaction	F_TS_ (cm^−1^)	κ	Q_A_	Q_B_	Q_TS_	ΔE (kcal/mol)	k (s^−1^/(mole/cm^3^))
①	−1994.99	4.871	6.38 × 10^12^	9.18 × 10^17^	2.39 × 10^21^	14.573	1.042 × 10^−26^
②	−1781.46	4.087	-	-	1.88 × 10^21^	8.786	1.208 × 10^−22^
③	−2036.75	5.504	-	-	9.95 × 10^21^	16.688	1.263 × 10^−27^
④	−2041.48	5.054	-	-	8.85 × 10^21^	16.975	6.934 × 10^−28^
⑤	−1565.33	3.383	-	-	1.22 × 10^21^	6.186	5.232 × 10^−21^
③′	−455.59	1.202	-	-	8.9 × 10^20^	11.632	1.375 × 10^−25^
④′	−449.13	1.196	-	-	8.37 × 10^20^	11.740	1.078 × 10^−25^

“-” represents that the value is the same as above.

**Table 2:** Changes in internal energy, enthalpy and Gibbs free energy of the reaction process.

Reaction	${\Delta U}_{298K}^{{}^{\circ}}$ (kcal/mol)	${\Delta H}_{298K}^{{}^{\circ}}$ (kcal/mol)	${\Delta G}_{298K}^{{}^{\circ}}$ (kcal/mol)
①	6.354	6.354	6.685
②	−12.518	−12.518	−10.535
③	11.995	11.995	13.311
④	12.568	12.568	13.735
⑤	−22.327	−22.327	−20.656
③′	−6.143	−6.735	7.823
④′	−9.614	−10.206	4.046

## Data Availability

All data generated or analyzed during this study are included in this published article.
